# Health quotient of puerperal women: a retrospective observational study

**DOI:** 10.3389/fpubh.2025.1537392

**Published:** 2025-08-06

**Authors:** Zhenzhen Wang, Rong Nie, Qian Zhang, Liping Zhou, Congcong Zhao, Xianghong Cheng

**Affiliations:** ^1^Department of Maternity, Henan Provincial People’s Hospital, People’s Hospital of Zhengzhou University, Zhengzhou, China; ^2^School of Health Science & Nursing, Wuhan Polytechnic University, Wuhan, China

**Keywords:** puerperium, women, health quotient, inventory survey, fluencing factors

## Abstract

**Introduction:**

The health quotient (HQ) represents health awareness, health knowledge, and health ability. There are few studies on the HQ of women in the puerperium.

**Methods:**

The convenience sampling method was used to select puerperium women.

**Results:**

A total of 245 questionnaires were valid. The average scores of health awareness, health knowledge, and health ability of women were 15.0 ± 1.6 (maximum: 18), 26.6 ± 7.9 (maximum: 42), and 25.8 ± 5.3 (maximum: 33) points, respectively. The residence of rural (OR = 3.489, *p* = 0.018) and whether the perinatal caregiver was a nanny (OR = 2.538, *p* = 0.019) were independently associated with health awareness. The occupational status of on-the-job (OR = 2.573, *p* = 0.010) and whether the payment method was medical insurance (OR = 0.233, *p* = 0.048) were independently associated with health knowledge. The place of residence (OR = 3.090, *p* = 0.040) and parity ≥ 2 births (OR = 4.324, *p* = 0.001) were independently associated with health ability. The structural equation model showed that the maternal health knowledge had direct effects on maternal health ability (*β* = 0.316, *p* = 0.011) and baby health ability (*β* = 0.327, *p* = 0.029), the baby health knowledge directly affected the baby health ability (*β* = 0.462, *p* < 0.001), and the health awareness directly affected the maternal health ability (*β* = 0.569, *p* = 0.006).

**Conclusion:**

Puerperium women had sufficient health awareness, suboptimal health knowledge, and suboptimal health ability. The residence, perinatal caregiver, occupational status, payment method, and parity were risk factors of HQ.

## Introduction

The outline of the “Healthy China 2030” strategy pointed out that “the health problems of key groups such as women, children, the older adult, and the disabled should be highlighted to improve the health of the whole people” (1–5). Pregnant women represent a special population. Ensuring their physical and mental health reflects the quality of women’s healthcare work in China and will have repercussions on the physical and mental health of the children and the next generation of workers. Hence, improving the care of pregnant women and their children is a key measure to improve the overall quality of the nation.

The health quotient (HQ) was derived and developed based on the concept of health, reflecting the level of health awareness, health knowledge, and health ability of individuals or residents of a certain area (6, 7). In this study, health awareness captures personal perceptions and attitudes toward health and well-being; health knowledge refers to the individual’s understanding of health-related information and disease management; and health ability indicates practical skills related to maternal self-care and neonatal care. These domains align with the conceptual framework proposed by Wan et al. (8, 9).

HQ, like the intelligence quotient and emotional quotient, represents the basic health literacy of individuals, including self-care, health knowledge, health maintenance, and other aspects (6, 7). An important aspect of HQ is that it can be improved through the proper interventions (10–12). Maintaining HQ is beneficial for the self-management of patients with chronic conditions (6, 13). Studies have shown that introducing the concept of HQ into routine health education and nursing can fully mobilize the patients’ initiative, improve treatment compliance, and help patients rebuild a healthy lifestyle to promote and maintain health effectively (10–12, 14, 15).

The puerperium refers to the period from the end of childbirth to the time when all body organs (except the breast) gradually return to their pre-pregnancy state, which generally takes 6–8 weeks (14). The puerperium is an important period for the recovery of maternal organs and a critical period for the healthy growth of newborns (14, 15). Women in the puerperium are under great psychological pressure and hormonal changes and are prone to a sense of a lack of competence in their role as mothers, which can have a huge adverse impact on parenting health and family relationships (16–18). The HQ level of women in the puerperium period is of great significance to the health management of infants and the rehabilitation of mothers after childbirth (8). Still, there are few studies on the HQ of women in the puerperium. A better understanding of the HQ of such women is necessary to improve postpartum care and management and the health of mothers and their children.

Therefore, this study examined the HQ level of puerperium women. The results should provide a theoretical basis for the future development of puerperium health education and related interventions.

## Materials and methods

### Study design and participants

This study is based on the master’s thesis of Wan Shenxian, which involved the development and initial validation of the Postpartum Women Health Quotient Scale (PWHQS) (9). The present study further examines the scale’s applicability in a broader sample of puerperal women and explores the associated influencing factors.

The cross-sectional study included puerperium women by convenience sampling in the author’s Hospital between September 2020 and December 2020. This study was reviewed by the Medical Ethics Committee of the author’s Hospital. All participants signed the study informed consent form.

The inclusion criteria were (1) puerperium women, (2) ≥ 18 years of age, (3) able to communicate in writing or language, express thoughts clearly, and cooperate in completing the questionnaire, and (4) patients and their families gave informed consent and participated voluntarily. The exclusion criteria were (1) serious concomitant diseases, such as acute respiratory failure or (2) those who requested participation termination during the investigation.

### Data collection

The general information questionnaire was designed by the research team to collect maternal age, education, occupational status, place of residence, income, healthcare payment method, preterm birth, parity, mode of delivery, complications during pregnancy, etc.

The HQ was assessed using the PWHQS scale, which includes 31 items covering five original subdimensions (9). In the original validation study, the scale demonstrated high internal consistency with a Cronbach’s *α* coefficient of 0.908 and strong content validity, with a scale-level content validity index (S-CVI) of 0.939 (9).

For the purposes of this study, the original five subdimensions were reorganized into three dimensions: health awareness (6 items), health knowledge (14 items, combining maternal and infant health knowledge), and health ability (11 items, combining maternal and infant health ability). The original item content remained unchanged. Each item is graded using a Likert-4 scoring. The score of each dimension is the sum of the corresponding item scores, and the total score of the scale is 93 points. Each dimension was divided into a high-score group and a low-score group according to the score (above or below the average for each dimension). A higher score represents higher HQ levels.

Before the formal survey, the convenience sampling method was adopted, and 30 puerperium women were selected for pre-survey. After a 2-week interval, the same survey method was used to conduct a second survey on the same group of subjects. Correlation analysis was used, the test–retest reliability of the two surveys was calculated, and the results showed that the *r* was 0.947. The Cronbach’s *α* coefficients of the total items of the questionnaire and health awareness, health knowledge, and health ability were 0.908, 0.914, 0.892, and 0.934, respectively, indicating that the questionnaire had good reliability.

### Quality control

The inventory survey was carried out in strict accordance with the research plan. The personnel participating in the questionnaire survey were uniformly trained. Before issuing the questionnaires, the research purpose, content, and filling requirements were explained to the research subjects, and the informed consent form was signed to ensure the confidentiality of the data. The investigators distributed the questionnaires in person and collected them on the spot. The input data were double-checked.

### Statistical analysis

SPSS 23.0 (IBM, Armonk, NY, USA) was used for data analysis. The Kolmogorov–Smirnov test was used for testing the continuous variables for normal distribution. The continuous variables were presented as means ± standard deviation. The categorical variables were expressed as *n* (%). The correlations between the dimensions of HQ were analyzed using the Spearman rank correlation. The influencing factors of each dimension of HQ were analyzed by multivariable logistic regression, and a forest plot was drawn. The results were expressed using the adjusted odds ratio (OR) and the corresponding 95% confidence interval (CI). A structural equation model (SEM) analysis was performed to examine how each dimension influenced each other; the hypotheses were that health knowledge directly influenced health awareness and ability, while health awareness directly influenced health ability. Two-sided *p*-values <0.05 were considered statistically significant.

## Results

### General information of the participants

A total of 250 questionnaires were distributed, 245 valid questionnaires were retrieved, and the effective retrieval rate was 98.0%. The participants were 28.9 ± 3.8 years old, 143 (58.4%) had undergraduate and junior college education, and 23 (9.4%) had a master’s degree or above. About 90 and 10% of puerperal lived in urban and rural areas, respectively, and 8.6 and 19.6% of newborns were born premature and post-term, respectively. There were 134 (54.7%) and 111 (45.3%) mothers who delivered spontaneously and by cesarean section, respectively. There were 28 cases of maternal complications during pregnancy, accounting for 11.4%. The average scores of health awareness, health knowledge, and health ability of all subjects were 15.0 ± 1.6, 26.6 ± 7.9, and 25.8 ± 5.3, respectively. The general information of the participants is shown in Table 1.

**Table 1:** General information of the participants.

Variables	Health awareness		Health knowledge		Health ability	
	Low (*n* = 149)	High (*n* = 96)	Low (*n* = 127)	High (*n* = 118)	Low (*n* = 135)	High (*n* = 110)
Age, years, mean ± SD	28.9 ± 3.9	28.9 ± 3.8	28.8 ± 3.8	29.0 ± 3.9	28.9 ± 3.5	28.9 ± 4.2
Education, *n* (%)						
Middle school and below	21 (14.1%)	14 (14.6%)	24 (18.9%)	11 (9.3%)	17 (12.6%)	18 (16.3%)
High school and technical secondary school	29 (19.4%)	15 (15.6%)	22 (17.3%)	22 (18.6%)	24 (17.7%)	20 (18.2%)
Undergraduate and junior college	88 (59.1%)	55 (57.3%)	72 (56.7%)	71 (60.2%)	78 (57.8%)	65 (59.1%)
Postgraduate and above	11 (7.4%)	12 (12.5%)	9 (7.1%)	14 (11.9%)	16 (11.9%)	7 (6.4%)
Occupation status, *n* (%)						
Unemployed	65 (43.6%)	36 (37.5%)	65 (51.2%)	36 (30.5%)	57 (42.2%)	44 (40.0%)
On the job	84 (56.4%)	60 (62.5%)	62 (48.8%)	82 (69.5%)	78 (57.8%)	66 (60.0%)
Place of residence, *n* (%)						
Urban	137 (91.9%)	83 (86.5%)	114 (89.8%)	106 (89.8%)	127 (94.1%)	93 (84.5%)
Rural area	12 (8.1%)	13 (13.5%)	13 (10.2%)	12 (10.2%)	8 (5.9%)	17 (15.5%)
Income, CNY, *n* (%)						
<2,000	5 (3.4%)	2 (2.1%)	2 (1.6%)	5 (4.2%)	4 (3.0%)	3 (2.7%)
2,000–3,999	41 (27.5%)	19 (19.8%)	33 (26.0%)	27 (22.9%)	29 (21.5%)	31 (28.2%)
4,000–5,999	58 (38.9%)	34 (35.4%)	44 (34.6%)	48 (40.7%)	48 (35.6%)	44 (40.0%)
6,000–7,999	13 (8.7%)	14 (14.6%)	14 (11.0%)	13 (11.0%)	15 (11.1%)	12 (10.9%)
≥ 8,000	32 (21.5%)	27 (28.1%)	34 (26.8%)	25 (21.2%)	39 (28.8%)	20 (18.2%)
Pattern of payment, *n* (%)						
Self-pay	62 (41.9%)	35 (37.6%)	55 (44.3%)	42 (35.6%)	51 (38.3%)	46 (42.6%)
Medical insurance	11 (7.4%)	5 (5.4%)	12 (9.7%)	4 (3.4%)	11 (8.3%)	5 (4.6%)
Maternity insurance	75 (50.7%)	53 (57.0%)	57 (46.0%)	72 (61.0%)	71 (53.4%)	57 (52.8%)
Premature delivery, *n* (%)						
Full-term delivery	103 (69.1%)	73 (76.0%)	88 (69.3%)	88 (74.6%)	95 (70.4%)	81 (73.6%)
Premature delivery	15 (10.1%)	6 (6.3%)	9 (7.10%)	12 (10.1%)	9 (6.6%)	12 (10.9%)
Post-term delivery	31 (20.8%)	17 (17.7%)	30 (23.6%)	18 (15.3%)	31 (23.0%)	17 (15.5%)
Parity, *n* (%)						
First delivery	112 (75.2%)	71 (74.0%)	98 (77.2%)	85 (72.0%)	113 (83.7%)	70 (63.6%)
Second delivery or more	37 (24.8%)	25 (26.0%)	29 (22.8%)	33 (28.0%)	22 (16.3%)	40 (36.4%)
Delivery mode, *n* (%)						
Spontaneous labor	90 (60.4%)	44 (45.8%)	64 (50.4%)	70 (59.3%)	76 (56.3%)	58 (52.7%)
Caesarean section	59 (39.6%)	52 (54.2%)	63 (49.6%)	48 (40.7%)	59 (43.7%)	52 (47.3%)
Pregnancy complications, *n* (%)						
Yes	20 (13.4%)	8 (8.3%)	17 (13.4%)	11 (9.3%)	16 (11.9%)	12 (10.9%)
No	129 (86.6%)	88 (91.7%)	110 (86.6%)	107 (90.7%)	119 (88.1%)	98 (89.1%)

### Correlation analysis between dimensions of health quotient

Spearman rank correlation was used to analyze the correlation between the various dimensions of HQ levels. There was a moderate correlation between health ability and health knowledge, while there was a weak correlation between health awareness and health knowledge, as well as between health awareness and health ability. To further explore subgroup differences, stratified Spearman correlation analyses were conducted. As shown in Table 2, women with a junior college education or above exhibited a stronger correlation between health ability and health knowledge (*r* = 0.621, *p* < 0.001) compared to those with a high school education or below (*r* = 0.494, *p* < 0.001). Similarly, urban residents demonstrated stronger interdimensional correlations than rural residents. The correlation between health ability and health knowledge was also stronger in women who delivered by cesarean section (*r* = 0.671, *p* < 0.001) compared to those with natural delivery (*r* = 0.532, *p* < 0.001). These findings are detailed in Table 2.

**Table 2:** Correlation analysis of various dimensions of health quotient levels.

Variables	Health awareness-health knowledge		Health awareness-health ability		Health ability-health knowledge	
	*r*	*p*	*r*	*p*	*r*	*p*
Education levels						
Middle school and below	0.322	0.004	0.395	<0.001	0.494	<0.001
Junior college and above	0.202	0.009	0.275	<0.001	0.621	<0.001
Place of residence						
Urban	0.221	0.001	0.279	<0.001	0.554	<0.001
Rural area	0.441	0.027	0.534	<0.001	0.632	0.006
Parity						
First delivery	0.219	0.003	0.291	<0.001	0.535	<0.001
Second delivery or more	0.332	0.008	0.316	0.012	0.595	<0.001
Delivery mode						
Spontaneous labor	0.243	0.005	0.395	<0.001	0.532	<0.001
Caesarean section	0.267	0.005	0.251	0.008	0.671	<0.001

### Risk factors of health quotient levels

**Figure 1:**
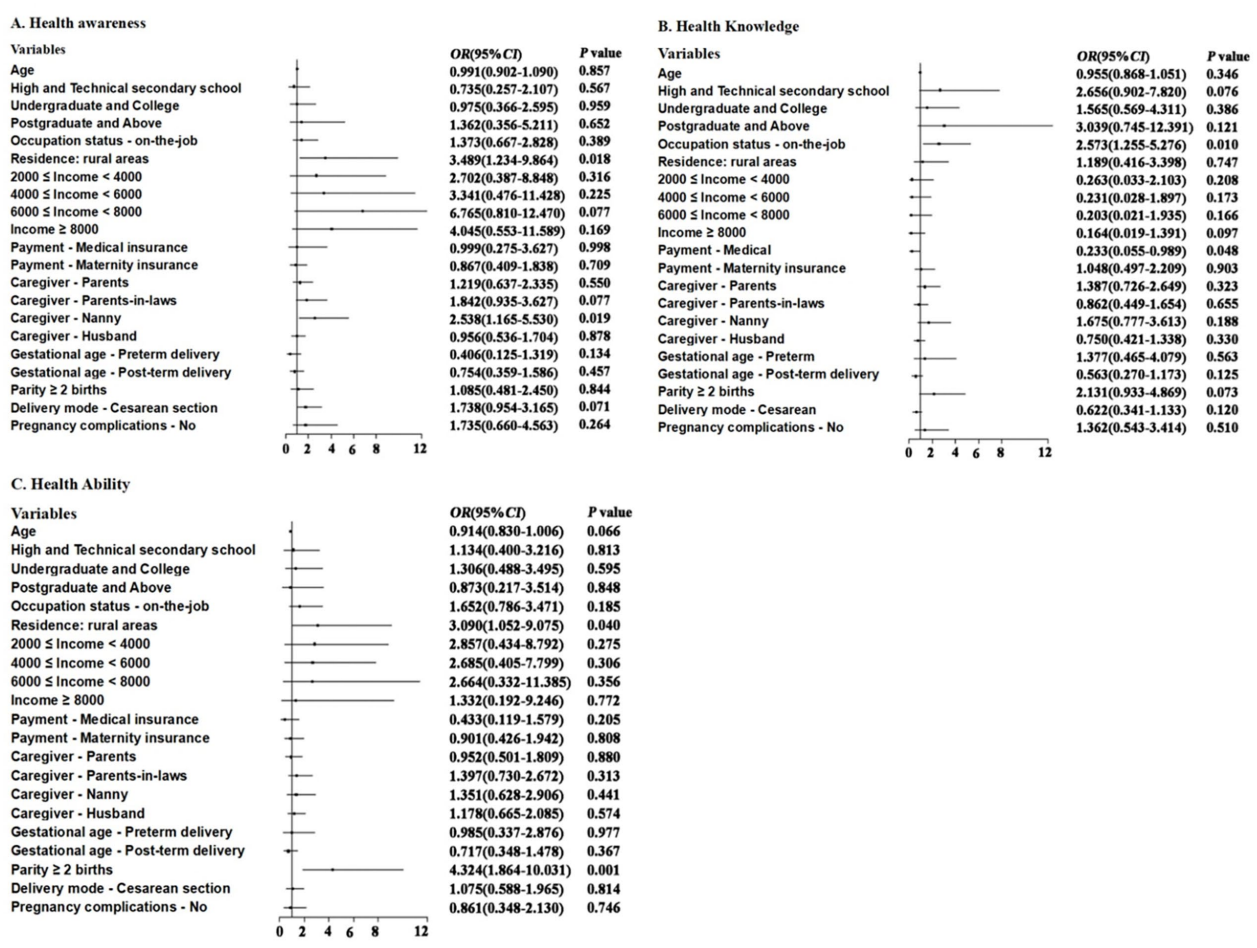
Forest plot of the influencing factors of **(A)** health awareness, **(B)** health knowledge, and **(C)** health ability.

The residence of rural (OR = 3.489, 95%CI: 1.234–9.864, *p* = 0.018) and whether the perinatal caregiver was a nanny (OR = 2.538, 95%CI: 1.165–5.530, *p* = 0.019) were independently associated with health awareness: mothers whose residence was in rural areas or whose caregiver was a nanny had lower health awareness. The occupational status of on-the-job (OR = 2.573, 95%CI: 1.255–5.276, *p* = 0.010) and whether the payment method was medical insurance (OR = 0.233, 95%CI: 0.055–0.989, *p* = 0.048) were independently associated with health knowledge: the puerperal on the job had lower health knowledge score, and the puerperal with the payment method of medical insurance had higher health knowledge score. The residence of rural (OR = 3.090, 95%CI: 1.052–9.075, *p* = 0.040) and parity ≥ 2 births (OR = 4.324, 95%CI: 1.864–10.031, *p* = 0.001) were independently associated with health ability: the puerperal living in rural areas had lower health ability score than those in urban areas, and the puerperal para >2 had lower health ability scores than women with a first parity (Figure 1).

### Structural equation model analysis

**Figure 2:**
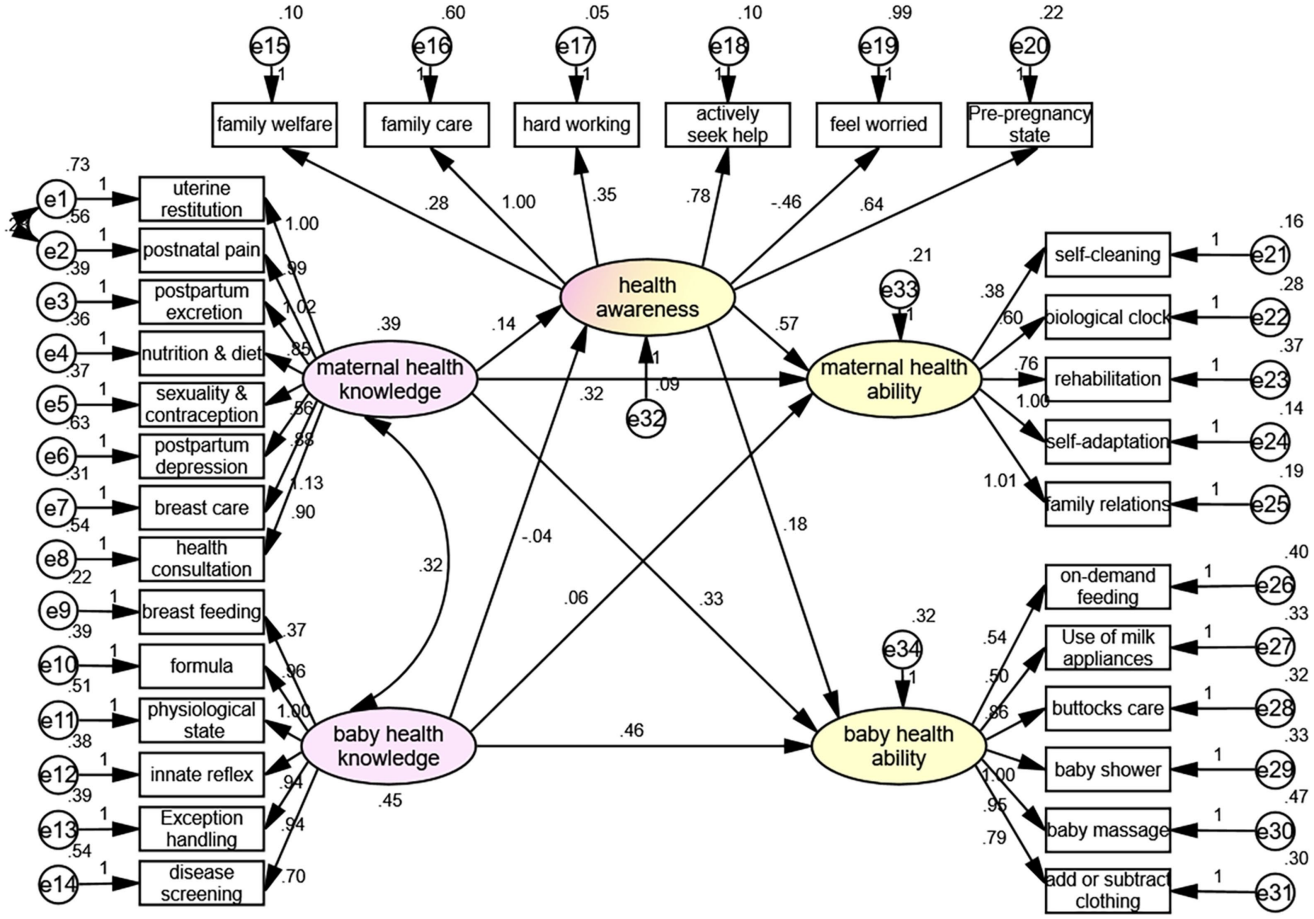
Structural equation model.

The SEM showed that the maternal health knowledge had direct effects on maternal health ability (*β* = 0.316, *p* = 0.011) and baby health ability (*β* = 0.327, *p* = 0.029), the baby health knowledge directly affected the baby health ability (*β* = 0.462, *p* < 0.001), and the health awareness directly affected the maternal health ability (*β* = 0.569, *p* = 0.006) (Figure 2, Table 3). Table 4 shows that the fit of the SEM was good.

**Table 3:** Structural equation model analysis results.

Variables		Variables	*β*	*p*
Health awareness	←	Maternal health knowledge	0.144	0.124
Health awareness	←	Baby health knowledge	−0.044	0.596
Maternal health ability	←	Maternal health knowledge	0.316	0.011
Baby health ability	←	Maternal health knowledge	0.327	0.029
Maternal health ability	←	Baby health knowledge	0.055	0.615
Baby health ability	←	Baby health knowledge	0.462	<0.001
Maternal health ability	←	Health awareness	0.569	0.006
Baby health ability	←	Health awareness	0.184	0.354

**Table 4:** Structural equation model analysis fit indicators.

Indicator	Reference standard	Measured results
CMIN/DF	<3, excellent	1.959
RMSEA	<0.08, good	0.063
IFI	>0.8, good	0.846
TLI	>0.8, good	0.828
CFI	<0.08, good	0.843

## Discussion

The results suggested that the HQ of puerperal women was intermediate, consistent with previous findings indicating that health literacy among pregnant women in China remains suboptimal (18–22). The present study identified key risk factors affecting HQ, including residence, perinatal caregiver type, occupational status, payment method, and parity. These findings align with previous research using the original PWHQS, which also emphasized the significant influence of socioeconomic factors on HQ levels (18, 23).

Most women have insufficient or lack maternal and child healthcare knowledge and nursing ability during the puerperium, especially primiparas, whose poor physical condition, hormonal changes, and psychological problems seriously affect their health and can also bring negative effects on the growth and development of newborns (15–17, 19, 20). The present study showed that the levels of health awareness, health knowledge, and health ability were interrelated, and positive correlations existed among these dimensions. Specifically, health knowledge was found to directly affect both maternal health ability (*β* = 0.316, *p* = 0.011) and baby health ability (*β* = 0.327, *p* = 0.029). This finding is in line with Wang Zhenzhen, who reported that infant-related health knowledge positively influences puerperal women’s HQ through infant-related health ability (19).

Furthermore, our study revealed that health awareness also directly influenced maternal health ability (*β* = 0.569, *p* = 0.006), a finding that extends previous literature by emphasizing the importance of health awareness in maternal self-care capacity. However, the correlation between health awareness and health knowledge was relatively weak, indicating that having health knowledge does not necessarily translate into heightened health awareness, particularly among women with lower educational backgrounds.

The residence of rural areas and having a nanny as a perinatal caregiver were independently associated with lower health awareness. This finding is consistent with Wan et al. and Wang Rong, who also found that urban residency and higher educational attainment are associated with better HQ levels (9, 23). The lower health awareness among women with nannies may reflect the delegation of maternal responsibilities to caregivers, potentially reducing the mother’s health management engagement.

In contrast, our study uniquely identified occupational status as a significant factor affecting health knowledge. Employed women demonstrated lower health knowledge scores compared to unemployed women, which may be attributable to the limited time and resources available for health education among working women. Previous studies have not emphasized this association, suggesting a new perspective for targeting health education interventions for employed mothers. Moreover, the present study highlighted that the payment method also impacted health knowledge. Women using medical insurance exhibited higher health knowledge scores, possibly due to greater access to health resources and educational materials associated with insurance coverage. This is a novel finding, as previous research primarily focused on educational and social factors without considering economic access as a determinant of health knowledge.

Parity was another significant factor affecting health ability, with women having two or more births showing lower health ability scores compared to primiparas. This is contrary to Wang et al., who reported that increased parity was associated with higher self-care ability due to accumulated maternal experience (23). However, our findings suggest that multiple births may impose a heavier physical and psychological burden on mothers, potentially reducing their capacity for effective health management.

The identification of risk factors and the interrelationship among health awareness, knowledge, and ability provide valuable insights for targeted health education programs. To improve HQ levels, specific attention should be given to women in rural areas, those with multiple births, and working mothers, as these subgroups are more likely to exhibit lower HQ scores. Additionally, integrating HQ-focused health education into digital platforms and community outreach programs could further enhance health literacy among postpartum women (24).

This study had limitations. Participants were recruited from a single tertiary hospital using a convenience sampling method, which may limit the generalizability of the results and introduce potential selection bias. Future studies should consider recruiting larger, more diverse samples across multiple centers to enhance external validity. Psychological factors such as depression and anxiety, which are common during the postpartum period and may influence health behaviors and self-management ability, were not assessed in this study. The absence of such measures may limit a more comprehensive understanding of the determinants of HQ. Future studies should consider incorporating validated psychological assessments to explore the potential mediating or moderating effects of mental health on HQ levels. Additional longitudinal data will also be helpful in identifying temporal relationships and designing more targeted interventions.

## Conclusion

The health knowledge, health awareness, and health ability of puerperium women were positively correlated. The HQ levels of women during the puerperium were affected by the place of residence, caregiver, employment status, payment method, and parity. The SEM showed that maternal health knowledge directly affected maternal health ability and baby health ability, baby health knowledge directly affected the baby health ability, and health awareness directly affected the maternal health ability. Hence, improving knowledge is important.

## Data Availability

The original contributions presented in the study are included in the article/supplementary material, further inquiries can be directed to the corresponding author.
